# Creation of Standardized Common Data Elements for Diagnostic Tests in Infectious Disease Studies: Semantic and Syntactic Mapping

**DOI:** 10.2196/50049

**Published:** 2024-06-10

**Authors:** Caroline Stellmach, Sina Marie Hopff, Thomas Jaenisch, Susana Marina Nunes de Miranda, Eugenia Rinaldi, The NAPKON, LEOSS, ORCHESTRA, and ReCoDID Working Groups

**Affiliations:** 1 Berlin Institute of Health Charité - Universitätsmedizin Berlin Berlin Germany; 2 Center for Integrated Oncology Aachen Bonn Cologne Duesseldorf, Department I of Internal Medicine University Hospital Cologne and Faculty of Medicine University of Cologne Cologne Germany; 3 Heidelberg Institut für Global Health Universitätsklinikum Heidelberg Heidelberg Germany; 4 See Acknowledgments

**Keywords:** core data element, CDE, case report form, CRF, interoperability, semantic standards, infectious disease, diagnostic test, covid19, COVID-19, mpox, ZIKV, patient data, data model, syntactic interoperability, clinical data, FHIR, SNOMED CT, LOINC, virus infection, common element

## Abstract

**Background:**

It is necessary to harmonize and standardize data variables used in case report forms (CRFs) of clinical studies to facilitate the merging and sharing of the collected patient data across several clinical studies. This is particularly true for clinical studies that focus on infectious diseases. Public health may be highly dependent on the findings of such studies. Hence, there is an elevated urgency to generate meaningful, reliable insights, ideally based on a high sample number and quality data. The implementation of core data elements and the incorporation of interoperability standards can facilitate the creation of harmonized clinical data sets.

**Objective:**

This study’s objective was to compare, harmonize, and standardize variables focused on diagnostic tests used as part of CRFs in 6 international clinical studies of infectious diseases in order to, ultimately, then make available the panstudy common data elements (CDEs) for ongoing and future studies to foster interoperability and comparability of collected data across trials.

**Methods:**

We reviewed and compared the metadata that comprised the CRFs used for data collection in and across all 6 infectious disease studies under consideration in order to identify CDEs. We examined the availability of international semantic standard codes within the Systemized Nomenclature of Medicine - Clinical Terms, the National Cancer Institute Thesaurus, and the Logical Observation Identifiers Names and Codes system for the unambiguous representation of diagnostic testing information that makes up the CDEs. We then proposed 2 data models that incorporate semantic and syntactic standards for the identified CDEs.

**Results:**

Of 216 variables that were considered in the scope of the analysis, we identified 11 CDEs to describe diagnostic tests (in particular, serology and sequencing) for infectious diseases: viral lineage/clade; test date, type, performer, and manufacturer; target gene; quantitative and qualitative results; and specimen identifier, type, and collection date.

**Conclusions:**

The identification of CDEs for infectious diseases is the first step in facilitating the exchange and possible merging of a subset of data across clinical studies (and with that, large research projects) for possible shared analysis to increase the power of findings. The path to harmonization and standardization of clinical study data in the interest of interoperability can be paved in 2 ways. First, a map to standard terminologies ensures that each data element’s (variable’s) definition is unambiguous and that it has a single, unique interpretation across studies. Second, the exchange of these data is assisted by “wrapping” them in a standard exchange format, such as Fast Health care Interoperability Resources or the Clinical Data Interchange Standards Consortium’s Clinical Data Acquisition Standards Harmonization Model.

## Introduction

In response to the spread of SARS-CoV-2 starting in late 2019, large-scale observational studies as well as clinical trials have been launched worldwide to gain insights into disease patterns, treatment options, prevention measures, severity, and outcomes [1]. New findings related to the diagnosis, prevention, and treatment of many infectious diseases, including COVID-19, heavily rely on data generated by diagnostic tools and laboratory analysis of the pathogen and host response [2].

Immunological testing has become a cost- and time-efficient way to monitor infections [3]. Hence, a growing number of clinical studies include biosample information as part of their data collection targets, particularly results of analytical tests performed on blood samples [4].

Data from patients enrolled in a study are commonly collected using a case report form (CRF) [5]. The International Conference on Harmonization Guidelines for Good Clinical Practice defines a CRF as a “printed, optical or electronic document designed to record all of the protocol-required information to be reported to the sponsor on each trial subject” [6]. Since the design of a CRF can affect study outcomes, time and resources need to be invested to maximize the quality of the data collected and ensure that good clinical practice guidelines are being followed [7].

The identification of common data elements (CDEs), each comprising 1 or more questions and respective answer value sets, is an approach to standardize data collection instruments (ie, CRFs) across studies [8]. A CDE may also contain standardized ontology concepts directly or include a link to the unique identifier for an appropriate ontology concept [9].

We have previously described [10] how incorporating standard codes into clinical trials metadata can increase their findability, accessibility, interoperability, and reusability (FAIR)ness [11]. The FAIR principles are recognized internationally as important guides to conducting research [12]. Interoperability, in particular, is defined as the ability of several systems to exchange information, as well as read and use the received information without requiring further preprocessing [13]. Although there are several levels of interoperability [14], the focus of this study in the context of health care data was on semantic (use of standard terminologies and classifications) and syntactic (implementation of a standard exchange format) interoperability.

The use of data standards when designing CRFs can serve multiple purposes: in addition to supporting data quality, it facilitates the merging and exchange of data from multiple sources, as well as subsequent analysis [5]. International standards development organizations (SDOs), such as Health Level Seven (HL7) or Integrating the Healthcare Enterprise (IHE), promote and coordinate the use of these standards [15]. HL7 has developed the exchange standard Fast Healthcare Interoperability Resource (FHIR), which allows for the exchange of health-related information based on packaging it into so-called resources. The FHIR can represent a wide range of data, particularly those generated in care settings [16]. In comparison, the Clinical Data Interchange Standards Consortium (CDISC) has published standards for the representation of CRF data used in clinical trials [17].

By mapping study data elements to international semantic standard codes, the included concepts receive an unambiguous definition that is tied to an identifier that makes it machine-readable [18]. Among the widely used terminologies and classifications for health care concepts are the Logical Observation Identifiers Names and Codes (LOINC) and the Systematized Nomenclature of Medicine – Clinical Terms (SNOMED CT). The National Cancer Institute Thesaurus (NCIt) is also available as a reference terminology focused, among others, on translational research and clinical care information [19]. LOINC provides standard codes (each comprising a set of an identifier, a name, and a code) for laboratory observations, documents, and questionnaires [20]. SNOMED CT covers a broad range of health care information, and each of its concepts has a unique identifier and is defined by a description and 1 or more relationships [21].

In this study, we set out to analyze CRF variables from 6 study protocols capturing information about diagnostic testing with the purpose of identifying CDEs specific to infectious diseases. The selected studies investigated 3 different infectious diseases in humans: COVID-19 [1], monkeypox (mpox) [22], and Zika [23]. The CRF variables we included originate from the International Severe Acute Respiratory and emerging Infection Consortium (ISARIC) COVID-19 Core CRF [24], as well as from 3 of the many international research projects focused on gaining new insights into SARS-CoV-2: the ORCHESTRA project [25], the Intersectoral Platform (SUEP) of the National Pandemic Cohort Network (NAPKON SUEP) study [26], and the Lean European Open Survey on SARS-CoV‑2 (LEOSS) [27] study. Additionally, we analyzed the World Health Organization (WHO) CRF on the mpox infection [28] and the Zika CRFs of ZIKAlliance [29].

Our goal of proposing standardized paninfectious disease CRF variables for diagnostic testing information for use in CRFs was broken down into 3 subtasks: (1) identification of interstudy CDEs, (2) creation of a preliminary map of the CDEs to semantic standard codes, and (3) development of a proposed mapping of the CDEs to the FHIR syntax standards [30] and the CDISC’s standards for data collection [17].

## Methods

### Ethical Considerations

Since only CRF metadata (meaning definitions of questions and answers used to comprise CRFs) were used and no actual patient data were reviewed in this study, ethics approval was not required.

### Study Design

**Figure 1:**
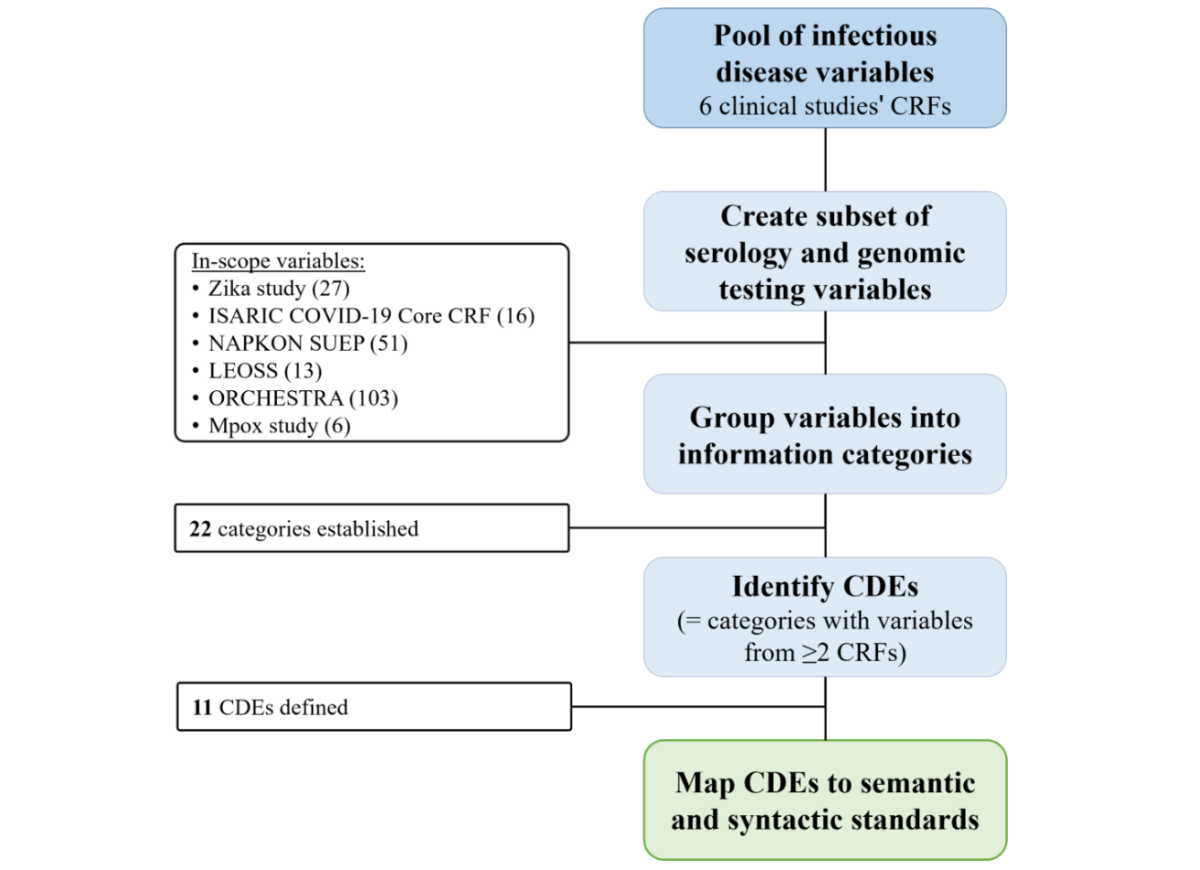
Flowchart describing the methodology of identifying common, standardized CRF variables reporting on diagnostic testing for use in infectious disease studies. CDE: common data element; CRF: case report form; ISARIC: International Severe Acute Respiratory and emerging Infection Consortium; LEOSS: Lean European Open Survey on SARS-CoV 2; mpox: monkeypox; NAPKON SUEP: Intersectoral Platform (SUEP) of the National Pandemic Cohort Network.

Figure 1 provides a graphical overview of the steps we followed to create a standardized set of variables for use in data collection instruments in infectious disease studies focusing on diagnostic testing.

We examined 6 CRFs provided to us by 4 research consortia, and we downloaded the publicly available CRFs from the ISARIC and WHO websites [24]. We proceeded to extract diagnostic testing variables from each CRF and organized them for analysis and comparison in a Microsoft Excel sheet.

The following CRFs were included:

- ORCHESTRA work package 6 CRF [31]
- Cross-sectoral platform (SUEP) CRF of NAPKON [32]
- LEOSS study [27,33] electronic case report form (eCRF)
- ISARIC-WHO COVID-19 core CRF [24]
- Zika study CRF
- Mpox study CRF [28]

We translated the variables from the NAPKON SUEP study from German into English to harmonize it with the language of the other selected studies (English). The study manager verified the translation.

### Common Data Elements

In the first step of analyzing the study metadata, we reviewed all CRF variables (questions and answers). Adopting the National Institutes of Health’s methodology to derive CDEs [34], we created common categories to group variables based on the key information they contained. We then reviewed the newly organized variables to determine which categories were present in at least 2 (33%) of the 6 CRFs. These common variables then formed the basis as newly identified CDEs for infectious diseases.

For each of these preliminary CDEs, the extensive value set (sum of all unique answers) across all reviewed CRFs was determined. If necessary, we created value set subsets based on informational content and pathogen type.

### Mapping to Standards

Each CDE (question and value set) was then mapped to the appropriate semantic standard code(s) and FHIR element(s). We searched for available terminology codes using the NCIt browser (version 23.02d, release date February 27, 2023), the SearchLOINC tool (v2.26), and the SNOMED CT browser (version 2023-03-31). If no semantic standard code was found, we prepared a submission to request the creation of a new code, depending on the informational domain, with NCIt, SNOMED CT, or LOINC.

## Results

### CRF Analysis

The analysis of the CRFs used in 6 infectious disease studies led to the identification of 216 variables focusing on diagnostic testing, which were in the scope of further analysis: 103 (47.7%) from ORCHESTRA, 51 (23.6%) from NAPKON SUEP, 27 (12.5%) from the Zika study, 16 (7.4%) from the ISARIC CRF, 13 (6%) from the LEOSS survey, and 6 (2.8%) from the mpox study (Table S1 in Multimedia Appendix 1 [25,28,32,35-42]). These diagnostic testing variables could be grouped into 22 newly defined categories, which are shown in Table S2 in Multimedia Appendix 1.

### Common Data Elements

**Figure 2:**
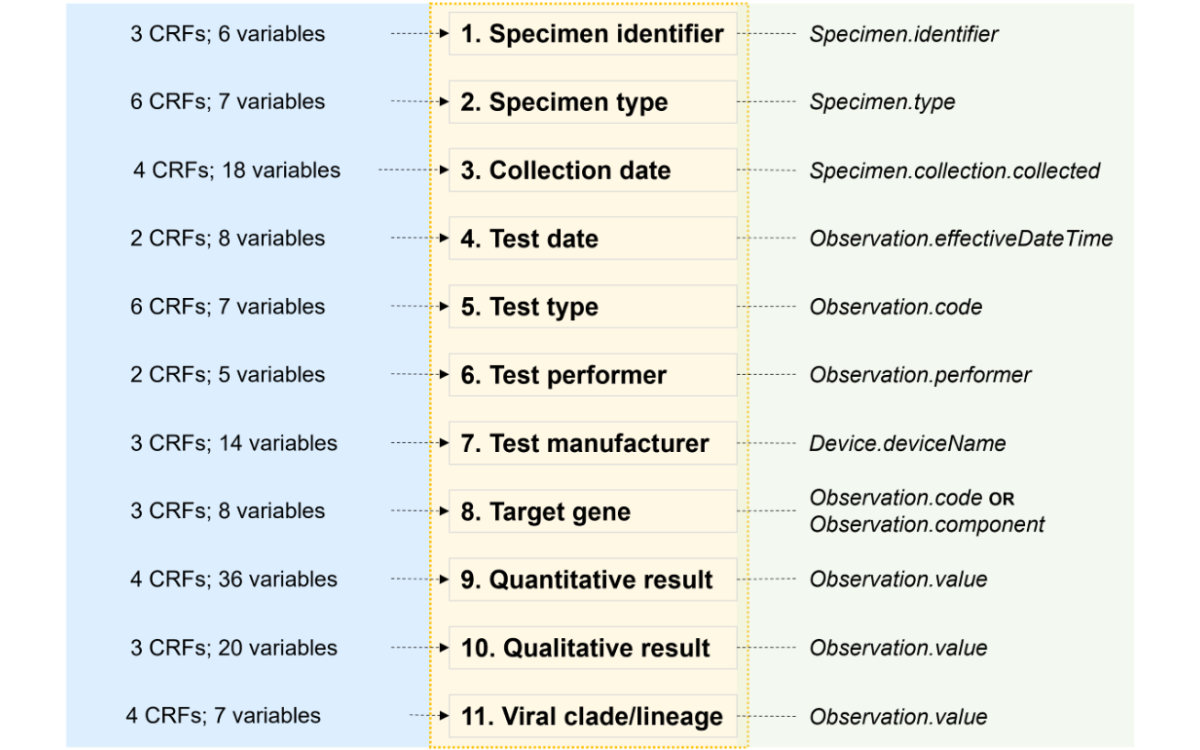
List of 11 CDEs identified based on the review of CRF variables from 6 infectious disease studies capturing diagnostic testing information. Also noted are the proposed data type and suggested mapping to the FHIR (version R4). For each CDE, the data type that was most commonly used across the reviewed data collection instruments is shown. CDE: common data element; CRF: case report form; FHIR: Fast Healthcare Interoperability Resources.

Based on the analysis of the 6 CRFs, we identified 11 CDEs, each of which was present in at least 2 (33%) of the 6 reviewed data collection instruments and reflected diagnostic testing information applicable to infectious disease studies. We mapped these CDEs to semantic standard codes and FHIR resources (illustrated in Figure 2), as well as to the CDISC (Multimedia Appendix 2).

#### Viral Lineage/Clade

**Figure 3:**
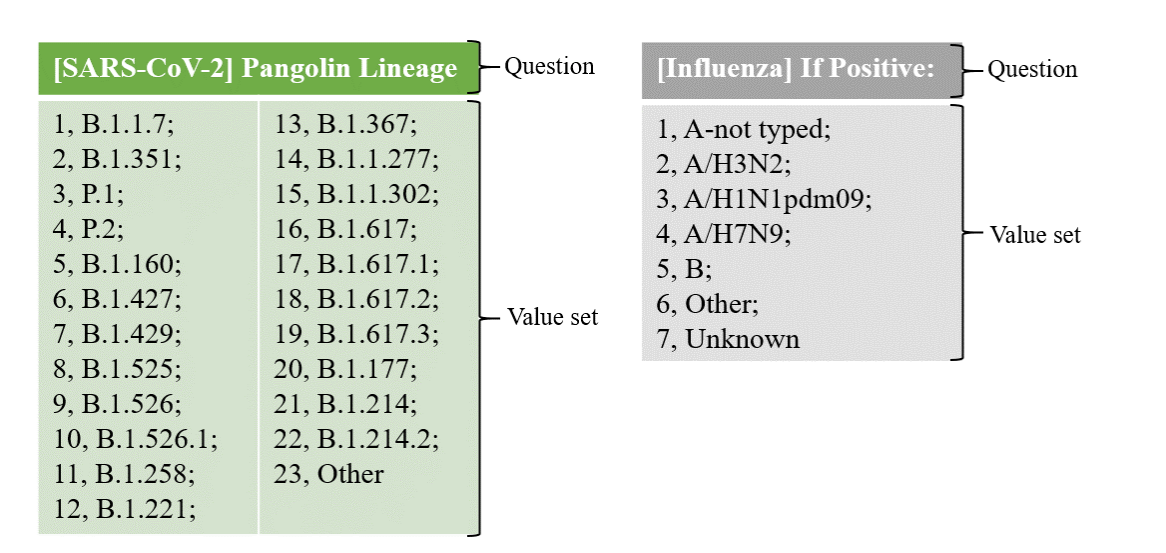
Two example variables that were grouped into the “viral lineage” CDE from two CRFs that were reviewed. CDE: common data element; CRF: case report form.

The first CDE was defined as “viral lineage” or “viral clade.” Depending on the virus investigated, its value sets would vary to reflect the applicable clade and lineage details, as exemplified in Figure 3.

Genetic diversity, as described in a phylogenetic tree, is classified by clades. A clade, also called genotype or subtype, comprises a set of lineages that are all descended from only 1 ancestor, common to them [43].

ORCHESTRA and the human mpox study contained 3 (1.4%) variables providing monkeypox virus (MPXV) and SARS-CoV-2 clade details. In addition, viral lineage information was collected from ORCHESTRA, the ISARIC CRF, and NAPKON SUEP across 4 (1.9%) variables.

There is no uniform convention for naming viral clades and lineages. In the case of SARS-CoV-2, the most widely used nomenclatures for subtypes are provided by the Global Initiative on Sharing All Influenza Data [44], Rambault et al [43], and Nextstrain [45], which differ in the position at which clades are differentiated from one another.

#### Specimen Identifier, Specimen Collection Date, and Specimen Type

Our analysis led to the identification of “specimen identifier,” “specimen collection date,” and “specimen type” as additional CDEs across the 6 studies. ORCHESTRA and the Zika and mpox studies included 6 (2.8%) variables that were grouped as “specimen identifiers” and had a free-text format. Any biological specimen (ie, blood, urine, cerebrospinal fluid, feces) used for laboratory analysis must be uniquely identified so that the resulting findings are associated with the right patient. Identifiers might contain a patient’s first and last names, birth date, medical facility number, or a unique, randomly generated code [46]. In addition to this internal laboratory-based specimen identifier, a particular specimen might have a second, external identifier that is assigned when results based on the analysis of said specimen are uploaded to a public/restricted databases or to a biobank [47].

Furthermore, 8 (3.7%) variables across the Zika study, NAPKON SUEP, ORCHESTRA, and ISARIC CRFs constituted the data element “specimen collection date,” requiring the input of a date format (mm/dd/yyyy). The specimen collection date marks the date on which a specimen was collected from a patient and placed in a specimen container for ensuing processing and analysis.

Details about the kind of specimen collected and used for analysis are provided by the coded “specimen type” CDE. All 6 reviewed studies included the data element “specimen type” in their variables. Our analysis led to the finding that there tended to be 2 axes involved in the value set elements of the specimen type, which covered information about the method used to collect the specimen (ie, swab) and the site of origin (ie, skin lesion). Examples are shown in Multimedia Appendix 3.

#### Test Date and Test Performer

The CDEs “test date” and “test performer” included variables from the ORCHESTRA and Zika study CRFs and the ORCHESTRA and NAPKON SUEP CRFs, respectively. The test date refers to the calendar date on which a particular laboratory diagnostic test (specified by the CDE “test type”) was conducted. The “test performer” CDE captures the full name of the individual(s) executing this diagnostic test in free-text format.

#### Test Type, Target Gene, and Test Manufacturer

The coded CDE “test type” captures a specific laboratory test, which in this context would fall into 3 main categories: serology, sequencing, and polymerase chain reaction (PCR) analysis. All 6 reviewed CRFs included variables providing details about diagnostic tests. For serology tests, the test type in the analyzed SARS-CoV-2 studies provided details on the method, along with the analyzed target, whereas in the Zika study, only the target was given (Multimedia Appendix 4).

In the context of COVID-19 research, lateral flow testing, immunofluorescence assay (IFA), enzyme-linked immunosorbent assay (ELISA), and chemiluminescence immunoassay (CLIA) are frequently used methods for the diagnosis of infections [48]. The detection of Zika and mpox infections is usually also based on serology, specifically ELISA-based antibody measurements [23].

The coded CDE “target gene” grouped 8 (3.7%) variables across the NAPKON SUEP, LEOSS, and ORCHESTRA CRFs. It refers to the target of a genome-focused diagnostic test, such as PCR or a sequencing method. Using primers that contain bases that are complementary to a conserved sequence within the target gene of a particular virus, this sequence, if present in the biological sample, is amplified and can be detected through PCR [49].

In total, 13 (6%) variables used across the Zika, NAPKON SUEP, and ORCHESTRA CRFs were grouped into the coded CDE “test manufacturer.” This data element provides information about the manufacturer of the diagnostic test (ie, kit or testing system). For example, the following PCR systems (manufacturers) were mentioned in a study variable in the NAPKON SUEP CRF: Seegene (Allplex) [50], altona Diagnostics (RealStar) [51], and Roche Deutschland Holding (cobas) [52].

#### Qualitative and Quantitative Results

All reported results of diagnostic testing covered by variables in the 6 CRFs we reviewed could be clustered into either qualitative or quantitative results, and thus, they formed the last 2 (18%) of 11 coded CDEs that we identified. A qualitative result details the findings about the presence or absence of a measured observable, such as virus-specific antibody or gene material. In contrast, a quantitative result constitutes numeric measurements (see Table S3 in Multimedia Appendix 1). In the studies that we analyzed, those numeric values were given for the titer, cycle threshold, and concentration of the same observables mentioned before.

### Mapping to Standards

#### Semantic Standards

To facilitate semantic interoperability of the proposed diagnostic testing CDEs, we suggested mapping each CDE and respective value set to the terminology standards SNOMED CT, LOINC, and NCIt. For each CDE, we created a suggested mapping that covers the variable itself and a nonexclusive list of possible value set elements (Table S4 in Multimedia Appendix 1).

The CDEs “viral lineage” and “viral clade” could be mapped to the following NCIt codes (code and description are shown), respectively: “C60792 Lineage” and “C179767 Clade.” Depending on the analyzed virus, the value sets (answers) could differ and be represented through mapping to either NCIt or LOINC codes. For example, in the case of detection of the SARS-CoV-2 variant B.1.1.7, the NCIt code “C179573 SARS Coronavirus 2 B.1.1.7” or the LOINC code “LA31705-9 SARS-CoV-2 B.1.1.7 lineage” is available.

The CDE “specimen identifier” could be represented in a standardized way using SNOMED CT, LOINC, and NCIt terms, as shown in Table S4 in Multimedia Appendix 1. Likewise, codes from all 3 standards were available to represent the free-text CDEs “specimen collection date” and “specimen type.”

There are semantic standard concepts available to describe the “test date” and “test performer” CDEs. Using SNOMED CT codes from the “procedure” hierarchy or using NCIt terms, diagnostic test types, such as serology assays, sequencing, and PCR, can be described in a standardized manner. Incidentally, there are a few standard codes available to represent the value sets for “target gene” (for the envelope gene in SNOMED CT and a few in the NCIt), although not necessarily specifically meant to map viral pathogens’ genes (exception in the NCIt: “C19108 Viral Envelope Gene”). Thus, we prepared a submission to the NCIt for the creation of concepts that cover the prominently analyzed SARS-CoV-2 [53] and Zika virus (ZIKV) genes [54]. We submitted 33 concepts for code creation to the SDOs LOINC and NCIt (Table S5 in Multimedia Appendix 1).

No SNOMED CT codes were available to describe the value set elements for the “test manufacturer” CDE. However, both the NCIt and LOINC provide terms for this purpose; the NCIt has created concepts for specific COVID-19 diagnostic kits, detailing the manufacturer, analytical target, and method. Likewise, LOINC has created codes that bundle several kits into a single term, such as “94558-4 SARS-CoV-2 (COVID-19) Ag [Presence] in Respiratory specimen by Rapid immunoassay,” which represents 4 commercially available kits [55].

There are generic semantic terms from SNOMED CT and the NCIt to describe the “quantitative result” and “qualitative result” CDEs in a standardized manner, which can be used across viral pathogen studies, such as “Laboratory Test Result” or just “Result.” However, this would omit the distinction between “qualitative” and “quantitative.”

LOINC provides a comprehensive list of terms to describe qualitative results of laboratory diagnostic tests for SARS-CoV-2 and antibody measurements specific to ZIKV.

The use of the SNOMED CT terminology requires a country (or institutional) license. SNOMED International has, however, been releasing its Global Patient Set containing currently around 24,000 concepts, which can be used free of charge [56]. Of the 90 SNOMED CT codes, 33 (37%) that we included in the exemplary value set mappings for our proposed infectious disease diagnostic CDEs are covered by the Global Patient Set.

### Syntax Standard

We proposed a preliminary mapping of the diagnostic testing CDEs to FHIR (version R4) elements as a first step toward establishing syntactical interoperability (Figure 2, right). Of the 11 CDEs that we identified, 8 (72.7%) were mapped to the *Observation* resource and the remaining 3 (27.3%) to the *Specimen* resource.

Additionally, we provided a preliminary suggested mapping of the FHIR elements to the CDISC according to the *FHIR to CDISC Joint Mapping Implementation Guide v1.0* [57] (see Multimedia Appendix 2).

## Discussion

### Principal Findings

#### Common Data Elements

Resulting from the review of 6 CRFs, we identified 11 panstudy CDEs that capture key diagnostic testing information commonly collected across the reviewed infectious disease studies. These CDEs were purposefully kept generic to enhance the probability that they could be adopted by researchers and integrated into data collection instruments of other infectious disease studies, even if a different pathogen was studied. The pathogen under investigation in a given study would determine the value set elements of CDEs of the coded data.

The CDEs “viral lineage” and “viral clade” provide the means to describe genetic relatedness of viruses, which is critical to pathogen surveillance and relies on the availability of well-defined nomenclature [58]. Currently, no panvirus approach to naming viral clades and lineages exists. The International Committee on Taxonomy of Viruses, founded in 1966, has the goal to develop a taxonomy for viruses and establish names for viral taxa based on international agreement. However, the International Committee on Taxonomy of Viruses does not address the naming of viral clades and lineages [59]. In the context of ensuring that diagnostic testing results are linked to the right sample (specimen) and patient, the CDEs “specimen identifier,” “specimen collection date,” and “specimen type” are important parameters. Regarding the diagnostic test itself, documentation of the CDEs “test date” and “test performer” can help identify quality problems retrospectively. Diagnostic testing results can be split into the CDEs “qualitative result” and “quantitative result,” which would confirm the presence/absence of signs of a pathogen or numeric values of measured observables, such as antibody titers. The CDEs “test type,” “target gene,” and “test manufacturer” provide all complementary details to the diagnostic tests conducted. Along with the increasing inclusion of molecular testing variables in the study of infectious diseases, we expect that this number of recurring elements (which would be candidate CDEs) that describe diagnostic tests across different studies will continue to grow.

The power of research findings can be expanded through combining data from several clinical studies for analysis in an effort to create a larger data set. Without considering privacy or legal considerations, the basis for merging data from different sources is that the correct information (ie, data variables) is linked together to ensure accuracy and avoid misinterpretation. Defining standardized CDEs that serve as a common language across clinical studies is one way to approach this challenge [9]. Lin et al [5] described a similar approach of how CRF design can be optimized for data harmonization by creating a pool of reusable CDEs. There are numerous examples for the creation of CDEs for specific medical specialties and use cases, such as stroke trials [60], pregnancy pharmacovigilance [61], and COVID-19 [62]. This includes a set of CDEs on the quality of life in neurological disorders, as well as the PhenX Toolkit to capture key information on phenotypes [8].

#### Mapping to Standards

To facilitate interoperability of study data in particular, we proposed a mapping of the identified CDEs to semantic and syntactic standards. We also created a table with practical examples of available standard codes to identify value set concepts ambiguously for variables contained in CRFs from studies focused on SARS-CoV-2, ZIKV, and MPXV (see Table S4 in Multimedia Appendix 1).

In the past, we have described how semantic interoperability standard codes can be integrated directly into the study metadata to facilitate merging, sharing, and analysis of patient data that are being collected across several clinical studies and cohort types, where several methods for data storage and collection have been used [10]. Kush et al [9] and Kersloot et al [11], among others, have discussed the advantage of introducing interoperability standards prior to data collection rather than retrospectively with the aim to save time and other resources.

An important aspect of mapping study data to semantic standard concepts is choosing appropriate terminology. Although there is no universal guidance for this process, we can draw instructive conclusions from our attempt to propose a mapping for the CDEs we identified for which we searched within the LOINC, SNOMED CT and NCIt, terminologies.

The selection of semantic standards to represent CDEs and their value sets depends on the way the CDEs (and underlying CRF variables) are phrased with regard to the level of detail and the kind of information that are described. The category of information covered by a CRF variable is the first “filter” for finding the appropriate terminology. The NCIt, which is managed by the National Cancer Institute, focuses on providing a vocabulary for the cancer domain [63]; hence, it comprises many (gen)omics-related terms. Each NCIt term is represented by a code and a name and has several annotations [64].

In contrast, the LOINC coding system, which is published by the Regenstrief Institute, is used by numerous large laboratories and government agencies, such as the Centers for Disease Control and Prevention, to describe laboratory and clinical findings, as well as documents [65]. Although LOINC has a clear focus on representing laboratory terms, SNOMED CT terms have a broader coverage of information and are commonly used to represent clinical information in electronic health records [63]. SNOMED CT and th eNCIt both provide concepts that are suitable to describe variables and value sets if they are kept more generic in their wording. LOINC terms, in contrast, are specific and should only be used to represent questions, not value sets. Contrary to the NCIt, SNOMED CT comprises a limited set of concepts to describe genomic methods and results.

Unlike the use of LOINC and the NCIt, embedding SNOMED CT concepts into the metadata of research data requires a license. In recent years, many countries have purchased a SNOMED CT affiliate license or become a SNOMED CT member, including Germany, Spain, and Portugal [66].

The LOINC coding system includes suitable codes for several of the CDEs we defined. For example, we chose the concept “95609-4 SARS-CoV-2 (COVID-19) S gene [Presence] in Respiratory specimen by Sequencing” as 1 of the available standard terms for coding the “qualitative result” CDE. However, it also covers the “target gene” (S gene), “specimen type” (respiratory specimen), and “test type” (sequencing) CDEs. Another aspect that should be kept in mind, especially concerning selecting standard terms for the “quantitative result” CDE when used in a CRF, is that the units of the result should be clearly defined and match those of the standard term. Although each LOINC term has a defined unit, SNOMED CT concepts do not necessarily implicitly or explicitly define units. The concept “1240461000000109 Measurement of severe acute respiratory syndrome coronavirus 2 antibody (observable entity)” has no unit of measure attached and hence can be used if a CRF variable can be measured using several different units. A standard way to describe units is offered by the Unified Code for Units of Measure [67].

Regarding finding the appropriate standard code for viral lineage, the more general-purpose terminology of SNOMED CT does not include the required level of detail for this CDE, which is captured in the NCIt. However, the list of microorganisms defined as concepts by SNOMED CT under the hierarchy “organism” is detailed and can be used to describe a pathogen. The hierarchical organization of SNOMED CT, which also includes sublevels of concepts, provides a clear idea of the positioning of any microorganism within the complex classification of organisms overall.

As knowledge rapidly evolves in health care, missing concepts are regularly added to ontologies. The process involves concept creation requests from the public, which are submitted to the SDOs. Zheng et al [63] describe an approach of using formal concept analysis to identify missing concepts in the NCIt and SNOMED CT.

We also proposed a mapping of the 11 diagnostic testing CDEs to the corresponding FHIR (version R4) element. This provides data with a standardized exchange format, which can incorporate standard terminologies. Elements in the *Specimen* [68] and *Observation* [69] (and for the test manufacturer, also *Device* [70]) resources can be used to represent all 11 CDEs.

### Limitations

The identified CDEs focus on diagnostic tests used in infectious disease studies. Additional CDEs that would fall into other informational categories (eg, therapeutics or comorbidities) were not considered as they were out of the scope of our study. Furthermore, since the reviewed ORCHESTRA variables include CRF variables from several COVID-19 studies, the selection of protocols might appear unbalanced.

### Conclusion

The need to investigate COVID-19 quickly and extensively has made the pool of available variables describing diagnostic tests particularly abundant. Kush et al [9] point out that although the name “CDE” implies that these elements are common, they are not so commonly used. This is due to a lack of mandatory requirements for their use [9]. A necessary step to increase the adoption and value of CDEs would be that funding bodies (eg, the National Institutes of Health or the European Commission) in collaboration with SDOs create and impose mandatory requirements for the implementation of existent CDEs on recipients of project funding.

## Appendix

Multimedia Appendix 1 Supplementary tables.

Multimedia Appendix 2 Map between the proposed FHIR representation (FHIR resources) and the corresponding CDISC elements for the 11 identified diagnostic testing CDEs for infectious disease studies. CDE: common data element; CDISC: Clinical Data Interchange Standards Consortium; FHIR: Fast Healthcare Interoperability Resources.

Multimedia Appendix 3 Example specimen types listed within CRF variables in the analyzed infectious disease studies. CRF: case report form.

Multimedia Appendix 4 Example variables (question and value set) from 2 of the reviewed CRFs. One came from the ZIKV study and the other from the NAPKON SUEP study. CLIA: chemiluminescence immunoassay; CRF: case report form; ELISA: enzyme-linked immunosorbent assay; IFA: immunofluorescence assay; IgA: immunoglobulin A; IgG: immunoglobulin G; IgM: immunoglobulin M; LFT: lateral flow immunoassay; PCR: polymerase chain reaction; SGTF: S gene target failure; VNTR: variable number of tandem repeats; WES: whole exome sequencing; WGS: whole genome sequencing; ZIKV: Zika virus.

## Abbreviations

CDE: common data element
CDISC: Clinical Data Interchange Standards Consortium
CRF: case report form
ELISA: enzyme-linked immunosorbent assay
FAIR: findability, accessibility, interoperability, and reusability
FHIR: Fast Healthcare Interoperability Resource
HL7: Health Level Seven
IHE: Integrating the Healthcare Enterprise
ISARIC: International Severe Acute Respiratory and emerging Infection Consortium
LEOSS: Lean European Open Survey on SARS-CoV‑2
LOINC: Logical Observation Identifiers Names and Codes
mpox: monkeypox
MPXV: monkeypox virus
NAPKON SUEP: Intersectoral Platform (SUEP) of the National Pandemic Cohort Network
NCIt: National Cancer Institute Thesaurus
PCR: polymerase chain reaction
SDO: standards development organization
SNOMED CT: Systematized Nomenclature of Medicine - Clinical Terms
WHO: World Health Organization
ZIKV: Zika virus

## Data Availability

The analyzed case report forms are stored and available in Excel format for review on the project’s online data repository [74].
