# Supplementary material for: Creation of Standardized Common Data Elements for Diagnostic Tests in Infectious Disease Studies: Semantic and Syntactic Mapping
Source: J Med Internet Res. 2024 Jun 10;26:e50049. doi: 10.2196/50049 (PMC11196918; doi:10.2196/50049)
Supplement: Multimedia Appendix 1 [file jmir_v26i1e50049_app1.docx]

Multimedia Appendix 1

# Background on sources of data used in this study

In the following, we provide a short overview of the project from which we received and analyzed case report forms.

## ISARIC COVID-19 CRF

A worldwide network of clinical research groups makes up the body of the International Severe Acute Respiratory and Emerging Infection Consortium (ISARIC). The ISARIC’s goal is to prevent illness and deaths from infectious diseases through collaboration and making research tools available to the public. In response to the COVID-19 pandemic and in collaboration with the World Health Organization (WHO), ISARIC published the ISARIC-WHO COVID-19 CRF online and for those who lacked adequate infrastructure for data collection, provided access to the data management platform REDCap[35], [36].

## LEOSS study

In order to record confirmed SARS-CoV-2 cases uniformly and to establish an evidence base for best practice in clinical management, analyses of a comprehensive SARS-CoV-2 cohort were required and mandated by the SARS-CoV-2 Emerging Infections Task Force (EITaF) of the European Society of Clinical Microbiology and Infectious Diseases (ESCMID) and the German Society of Infectious Diseases (DGI), in cooperation with the German Centre for Infection Research (DZIF). In this line, the project named Lean European Open Survey on SARS-CoV-2 Infected Patients (LEOSS) was initiated, which represented a prospective international multi-center cohort study an which aimed to get more in-depth knowledge about the epidemiology and clinical course of patients infected with SARS-CoV-2. For this project, an anonymous documentation approach was chosen, to be as time-efficient and comprehensive as possible and to include patients who would be unable to give their consent[37], [38].

## ZIKAlliance pregnant women and children cohort study

The ZIKAlliance consortium was funded by the European Commission (EC Horizon 2020) to investigate etiology and risk factors for congenital abnormalities in children born during the Zika virus epidemic in South America and the Caribbean. A multicentric birth cohort study was designed divided into a pregnant women (PW) cohort and a children (CH) cohort[39]. In addition, smaller history (NH) cohorts were initiated. Clinical cohort sites were selected over a broad geographic range within South America and the Caribbean.

## Mpox CRF

In response to the human monkeypox (mpox) outbreaks in 2022, the World Health Organization has developed and published a clinical characterization case report forms (CRF) to facilitate collection of data about clinical features of hospitalized patients and outpatients infected with mpox in a standardized manner[28].

## NAPKON SUEP study

The Cross-Sectoral Platform (SUEP) of the National Pandemic Cohort Network (NAPKON) was set up in Germany in November 2020. The study is part of the Network University Medicine (NUM) and was funded by the German Federal Ministry of Education and Research (BMBF) (FKZ: 01KX2021). The overall goal of NAPKON is to establish a harmonized, extensible, and interoperable network to support both the response to the current COVID-19 pandemic and its consequences, as well as future pandemics of any origin[32]. Within the NAPKON SUEP, data and bio samples from SARS-CoV-2 infected patients are prospectively collected and analyzed. The structure of the dataset is published on the NAPKON website[40].

## ORCHESTRA studies

The CRF variables we included from the European Commission funded ORCHESTRA project[25] were defined by work package (WP) 6 for use in the WP2 Long-Term Sequelae[41] and WP4 Fragile Population studies[42]. It focuses on laboratory tests conducted to diagnose presence of SARS-CoV-2 infection in enrolled patients.

# Supplementary Tables

| Table S1: Overview of case report forms that were analyzed as part of this study and the respective number of variables. |
| --- |
| \| **CRF Owner** \| **Number of included variables** \| \| --- \| --- \| \| ZIKAlliance PW CRF \| 27 \| \| ISARIC COVID-19 Core CRF \| 16 \| \| NAPKON SUEP study \| 51 \| \| LEOSS register \| 13 \| \| ORCHESTRA study \| 103 \| \| Mpox study \| 6 \| \| **Total** \| **216** \| |

| Table S2: Overview of the 21 defined variable categories that all CRF variables were grouped into. |
| --- |
| \| **Variable Categories** \| \| --- \| \| Collector \| \| Database version \| \| Genome assembly \| \| Interpretation \| \| Logic \| \| Microorganism \| \| OMICS method \| \| Other \| \| Qualitative result \| \| Quantitative result \| \| Sequencing platform \| \| Specimen ID \| \| Specimen type \| \| Specimen collection date \| \| Storage \| \| Target gene \| \| Test date \| \| Test manufacturer \| \| Test performer \| \| Test type \| \| Viral lineage / clade \| |

| Table S3: Tabular overview of the observables for which qualitative and quantitative results are recorded by the two respective CDEs and their value sets. |
| --- |
| \| **CDE** \| **Observable** \| \| **Value Set** \| \| --- \| --- \| --- \| --- \| \| **Qualitative Result** \| Antibody, Antigen, Gene \| \| detected / not detected / inconclusive \| \| positive finding / negative finding / inconclusive / weakly positive \| \|  \| \| \| \| \| **CDE** \| **Observable** \| \| **Value Set** \| \| **Quantitative Result** \| Antibody, Antigen, Gene \| titer \| numeric value \| |

| Table S4: Exemplary mapping of proposed common data elements for diagnostic testing in infectious disease studies to semantic standard terms from SNOMED CT, NCIt and LOINC. Font color indicates whether a term is specific to a viral pathogen: blue – Zika virus, green – SARS-CoV-2, orange – monkeypox virus, black – non-specific. |
| --- |
| \| **Common Data Element** \| **Type** \| **SNOMED CT terms  (Code and Fully Specified Name)** \| **NCIt (Code and Preferred Name)** \| **LOINC (Code and Long Common Name)** \| \| --- \| --- \| --- \| --- \| --- \| \| **1) Viral lineage / Viral clade** \| **Question coding** \| 50471002 Zika virus (organism) \| C14283 Virus \| 96755-4 SARS-CoV-2 (COVID-19) variant interpretation in Specimen Narrative \| \| 840533007 Severe acute respiratory syndrome coronavirus 2 (organism) \| C179767 Clade \| 96895-8 SARS-CoV-2 (COVID-19) lineage [Identifier] in Specimen by Molecular genetics method \| \| 59774002 Monkeypox virus (organism) \| C180323 Nextstrain Clade \| 96896-6 SARS-CoV-2 (COVID-19) clade [Type] in Specimen by Molecular genetics method \| \|  \| C60792 Lineage \| 96741-4 SARS-CoV-2 (COVID-19) variant [Type] in Specimen by Sequencing \| \|  \| C128553 Zika Virus \| 96755-4 SARS-CoV-2 (COVID-19) variant interpretation in Specimen Narrative \| \|  \| C184366 SARS-CoV-2 Variant of Concern \| 101003-2 Monkeypox virus clade [Type] in Specimen by Sequencing \| \|  \| C169076 SARS Coronavirus 2 \| 101004-0 Monkeypox virus lineage [Type] in Specimen by Sequencing \| \|  \| C187442 Monkeypox Virus \|  \| \| **Value set** \|  \| C184327 SARS Coronavirus 2 B.1.1.529 \| LA31569-9 SARS-CoV-2 Alpha variant (WHO) \| \| C180778 SARS Coronavirus 2 C.37 \| LA31570-7 SARS-CoV-2 Beta variant (WHO) \| \| C179585 SARS Coronavirus 2 B.1.617.1 \| LA31621-8 SARS-CoV-2 Gamma variant (WHO) \| \| C179586 SARS Coronavirus 2 B.1.617.2 \| LA32552-4 SARS-CoV-2 Delta variant (WHO) \| \| C179577 SARS Coronavirus 2 B.1.429 \| LA33381-7 SARS-CoV-2 Omicron variant (WHO) \| \| C179579 SARS Coronavirus 2 B.1.525 \| LA31705-9 SARS-CoV-2 B.1.1.7 lineage \| \| C179580 SARS Coronavirus 2 B.1.526 \| LA32553-2 SARS-CoV-2 B.1.351 lineage \| \| C179573 SARS Coronavirus 2 B.1.1.7 \| LA32555-7 SARS-CoV-2 P.1 lineage \| \| C179575 SARS Coronavirus 2 B.1.351 \| 100889-5 Monkeypox virus clade I DNA [Presence] in Specimen by NAA with probe detection \| \| C179599 SARS Coronavirus 2 P.1 \| 100888-7 Monkeypox virus clade II DNA [Presence] in Specimen by NAA with probe detection \| \| C179598 SARS Coronavirus 2 P.2 \|  \| \| **2) Specimen identifier** \| **Internal identifier** \| 372274003 Sample identification number (observable entity) \| C164332 Sample Identifier \| 89873-4 Unique identifier [Identifier] of Initial sample \| \|  \| C166393 Specimen Identifier \| 57723-9 Unique bar code number of Current sample \| \|  \|  \| 80398-1 Unique identifier for Current sample \| \| **External identifier** \|  \| C25402 Accession Number \| 87396-8 GenBank accession number \| \| C43685 GenBank Accession Number \| 96766-1 GISAID sequence accession number \| \| C180324 GISAID Accession ID \|  \| \| **3) Specimen collection date** \| **Question coding** \| 281271004 Date sample received in laboratory (observable entity) \| C178976 SARS-CoV-2 PCR Sample Collection Date \| 33882-2 Collection date of Specimen \| \| 168149003 Fluid sample collection time (observable entity) \| C81286 Collection Date \| 68963-8 Collection date and time of Specimen Collection date & time \| \| 399445004 Specimen collection date (observable entity) \| C178868 Biospecimen Collection Date \|  \| \| **Value set** \| 281326005 No specimen collection date given (finding) \|  \|  \| \| **4) Specimen Type** \| **Question coding** \| 371439000 Specimen type (observable entity) \| C70713 Biospecimen Type \| 66746-9 Specimen type \| \| 276823002 Type of specimen (attribute) \|  \|  \| \| **Value set** \| 258560004 Oral saliva specimen (specimen) \| C178987 Serum Sample \|  \| \| 441620008 Oral fluid specimen (specimen) \| C185204 Plasma Sample \|  \| \| 734846002 Semen component (substance) \| C189126 Urine Sample \|  \| \| 119347001 Seminal fluid specimen (specimen) \| C174119 Saliva Sample \|  \| \| 119373006 Amniotic fluid specimen (specimen) \| C13277 Semen \|  \| \| 122736005 Tissue specimen from placenta (specimen) \| C12692 Cerebrospinal Fluid \|  \| \| 258426009 Placental membrane tissue specimen (specimen) \| C185194 Cerebrospinal Fluid Sample \|  \| \| 258450006 Cerebrospinal fluid specimen (specimen) \| C13188 Amniotic Fluid \|  \| \| 57741000052105 Tracheal secretion specimen (specimen) \| C13272 Placenta \|  \| \| 445447003 Specimen from trachea obtained by aspiration (specimen) \| C17610 Blood Sample \|  \| \| 258469001 Pharyngeal washings (specimen) \| C158524 Skin Lesion \|  \| \| 309193002 Pharyngeal biopsy specimen (specimen) \| C189124 Sputum Sample \|  \| \| 258607008 Bronchoalveolar lavage fluid specimen (specimen) \| C189125 Stool Sample \|  \| \| 119307008 Specimen from endotracheal tube (specimen) \| C19697 Tissue Sample \|  \| \| 461911000124106 Swab specimen from oropharynx (specimen) \| C13190 Aqueous Humor \|  \| \| 258412000 Oropharyngeal aspirate (specimen) \| C18202 Biopsy Specimen \|  \| \| 1149105007 Oropharyngeal washings (specimen) \| C189123 Bronchoalveolar Lavage Specimen \|  \| \| 258500001 Nasopharyngeal swab (specimen) \| C171504 Endotracheal Fluid \|  \| \| 258467004 Nasopharyngeal washings (specimen) \| C155835 Oropharyngeal Swab Specimen \|  \| \| 258411007 Nasopharyngeal aspirate (specimen) \| C155831 Nasopharyngeal Swab Specimen \|  \| \| 122556008 Cord blood specimen (specimen) \| C155832 Nasopharyngeal Wash Specimen \|  \| \| 258436001 Umbilical cord tissue specimen (specimen) \| C120873 Cutaneous Crust \|  \| \| 119364003 Serum specimen (specimen) \| C32589 Female Genital System Fluid or Secretion \|  \| \| 119361006 Plasma specimen (specimen) \| C13713 Male Genital System Fluid or Secretion \|  \| \| 122575003 Urine specimen (specimen) \| C173641 Rectal Swab Specimen \|  \| \| 119342007 Saliva specimen (specimen) \| C13300 Umbilical Cord Blood \|  \| \| 258528007 Rectal swab (specimen) \| C34319 Umbilical Blood \|  \| \| 119297000 Blood specimen (specimen) \| C133318 Umbilical Cord Tissue \|  \| \| 309068002 Skin lesion specimen (specimen) \|  \|  \| \| 258603007 Respiratory specimen (specimen) \|  \|  \| \| 45710003 Sputum (substance) \|  \|  \| \| 258457009 Fecal fluid specimen (specimen) \|  \|  \| \| 119339001 Stool specimen (specimen) \|  \|  \| \| 119376003 Tissue specimen (specimen) \|  \|  \| \| 309128003 Eye fluid specimen (specimen) \|  \|  \| \| 258415003 Biopsy specimen (specimen) \|  \|  \| \| 445160003 Swab of eye (specimen) \|  \|  \| \| 258508008 Genital swab (specimen) \|  \|  \| \| **5) Test date** \| Question coding \| 439272007 Date of procedure (observable entity) \| C82512 Test Date \|  \| \|  \| C164023 Initial Genomic Sequencing Date \| \| **6) Test performer** \| Question coding \| 420158005 Performer of method (person) \| C48206 Performed By \| 68994-3 Performing laboratory name \| \| **7) Test type** \| Question coding \| 246246002 Test type (attribute) \| C173270 Laboratory Test Type \| 85069-3 Lab test method [Type] \| \| 246367000 Laboratory method (attribute) \| C83312 Laboratory Test Method \|  \| \| Value set \| 61594008 Microbial culture (procedure) \| C189805 Lateral Flow Assay \| 86205-2 Whole exome sequence analysis in Blood or Tissue by Molecular genetics method \| \| 30662005 Fluorescent immunoassay (procedure) \| C17370 Fluorescent Antibody Procedure \| 86206-0 Whole genome sequence analysis in Blood or Tissue by Molecular genetics method \| \| 76978006 Enzyme-linked immunosorbent assay (procedure) \| C16553 ELISA \| LA26398-0 Sequencing \| \| 1259935006 Chemiluminescent immunoassay technique (qualifier value) \| C111235 Chemiluminescent Immunoassay \| 101002-4 Monkeypox virus sequencing panel - Specimen by Sequencing \| \| 88667002 Viral serologic study (procedure) \| C120695 Virus Neutralization Assay \|  \| \| 47253003 Quantitative serology procedure (procedure) \| C17003 Polymerase Chain Reaction \|  \| \| 86810002 Qualitative serology procedure (procedure) \| C51962 Real Time PCR \|  \| \| 9718006 Polymerase chain reaction analysis (procedure) \| C127880 Nested PCR \|  \| \| 702675006 Probe with target amplification technique (qualifier value) \| C81971 Immunoglobulin G Measurement \|  \| \| 399150003 Polymerase chain reaction test for severe acute respiratory syndrome (procedure) \| C81972 Immunoglobulin M Measurement \|  \| \| 45293001 Immunoglobulin G measurement (procedure) \| C81969 Immunoglobulin A Measurement \|  \| \| 74889000 Immunoglobulin M (substance) \| C53465 Rapid Antigen Test \|  \| \| 31651002 Immunoglobulin A measurement (procedure) \| C18881 Nucleic Acid Sequencing \|  \| \| 121276004 Antigen assay (procedure) \| C101293 Next Generation Sequencing \|  \| \| 1156860005 Rapid antigen detection immunoassay technique (qualifier value) \| C186188 SARS-CoV-2 RdRp RNA Measurement \|  \| \| 117040002 Nucleic acid sequencing (procedure) \| C190874 Human Monkeypox Virus Measurement Finding \|  \| \| 115418004 Viral Sequencing (procedure) \|  \|  \| \| **8) Target gene** \| Question coding \| 67271001 Gene (substance) \| C16612 Gene \|  \| \| 3981005 Carrier detection molecular genetics (procedure) \|  \| \| Value set \| 7825004 ENV gene (observable entity) \| C19108 Viral Envelope Gene \|  \| \|  \| C26039 Membrane Protein Gene \| \|  \| C20744 Structural Protein Gene \| \| **9) Test manufacturer** \| Question coding \|  \| C180317 Nucleotide Sequencing Kit \| 74719-6 Manufacturer name \| \| C172274 Sequencing Platform Name \|  \| \| Value set \|  \| C180708 Abbott Architect SARS-CoV-2 QUANT II IgG Serology Assay; \| 94558-4 SARS-CoV-2 (COVID-19) Ag [Presence] in Respiratory specimen by Rapid immunoassay \| \| C180709 Roche Elecsys Anti-SARS-CoV-2 Serology Assay; \| *[Analysis of SARS-CoV-2 nucleocapsid protein antigen] includes manufacturers (kits):* \| \| C180725 DiaSorin LIAISON SARS-CoV-2 S1/S2 IgG Serology Assay; \| Abbott (BinaxNOW COVID-19 Ag Card) \| \| C180713 EUROIMMUN Anti-SARS-CoV-2 ELISA IgA Serology Assay; \| Access Bio Inc. (CareStart COVID-19 Antigen test*) \| \|  \| Becton Dickinson and Company (BD) (BD Veritor System for Rapid Detection of SARS-CoV-2*) \| \|  \| Luminostics Inc. (Clip COVID Rapid Antigen Test*) \| \|  \|  \| \| **10) Quantitative result** \| Question coding \| 767525000 Unit (qualifier value) \| C85776 Optical Density Measurement \| 55207-5 Genetic analysis discrete result panel \| \| 118289000 Optical density (observable entity) \| C44278 Unit \| 95427-1 SARS-CoV-2 (COVID-19) IgA Ab [Titer] in Serum or Plasma by Immunofluorescence \| \| 275924004 Result lab.- general (observable entity) \| C25709 Unit of Measure \| 95427-1 SARS-CoV-2 (COVID-19) IgA Ab [Titer] in Serum or Plasma by Immunofluorescence \| \| 275924004 Result lab.- general (observable entity) \| C36292 Laboratory Test Result \| 94510-5 SARS-CoV-2 (COVID-19) N gene [Cycle Threshold #] in Specimen by NAA with probe detection \| \| 68311008 Interferon assay (procedure) \| C161328 Threshold Cycle \| 94511-3 SARS-CoV-2 (COVID-19) ORF1ab region [Cycle Threshold #] in Specimen by NAA with probe detection \| \| 359897007 Anti-immunoglobulin A assay (procedure) \| C81969 Immunoglobulin A Measurement \| 94746-5 SARS-CoV-2 (COVID-19) RNA [Cycle Threshold #] in Specimen by NAA with probe detection \| \| 413027005 Immunoglobulin G autoantibody measurement (procedure) \| C81971 Immunoglobulin G Measurement \| 94643-4 SARS-CoV-2 (COVID-19) S gene [Cycle Threshold #] in Specimen by NAA with probe detection \| \| 250507002 Antibody titer measurement (procedure) \| C81972 Immunoglobulin M Measurement \| 94311-8 SARS-CoV-2 (COVID-19) N gene [Cycle Threshold #] in Specimen by Nucleic acid amplification using CDC primer-probe set N1 \| \| 1240461000000109 Measurement of severe acute respiratory syndrome coronavirus 2 antibody (observable entity) \|  \| 94312-6 SARS-CoV-2 (COVID-19) N gene [Cycle Threshold #] in Specimen by Nucleic acid amplification using CDC primer-probe set N2 \| \| 871560001 Detection of ribonucleic acid of severe acute respiratory syndrome coronavirus 2 using polymerase chain reaction (observable entity) \|  \| 94769-7 SARS-CoV-2 (COVID-19) Ab [Units/volume] in Serum or Plasma by Immunoassay \| \|  \|  \| 95410-7 SARS-CoV-2 (COVID-19) neutralizing antibody [Titer] in Serum by pVNT \| \|  \| 86321-7 Zika virus neutralizing antibody [Titer] in Specimen by Neutralization test \| \|  \| 89590-4 Zika virus IgG Ab [Titer] in Serum by Immunofluorescence \| \|  \| 89591-2 Zika virus IgM Ab [Titer] in Serum by Immunofluorescence \| \| **11) Qualitative result** \| Question coding \| 275924004 Result lab.- general (observable entity) \| C36292 Laboratory Test Result \| 55207-5 Genetic analysis discrete result panel \| \| 394617004 Result (navigational concept) \| C80324 Pathogen \| 60275-5 SARS coronavirus RNA [Presence] in Isolate by NAA with probe detection \| \| 3981005 Carrier detection molecular genetics (procedure) \| C178977 SARS-CoV-2 PCR Test Result \| 94309-2 SARS-CoV-2 (COVID-19) RNA [Presence] in Specimen by NAA with probe detection \| \| 871560001 Detection of ribonucleic acid of severe acute respiratory syndrome coronavirus 2 using polymerase chain reaction (observable entity) \|  \| 94641-8 SARS-CoV-2 (COVID-19) S gene [Presence] in Specimen by NAA with probe detection \| \| 399150003 Polymerase chain reaction test for severe acute respiratory syndrome (procedure) \|  \| 95609-4 SARS-CoV-2 (COVID-19) S gene [Presence] in Respiratory specimen by Sequencing \| \|  \|  \| 94639-2 SARS-CoV-2 (COVID-19) ORF1ab region [Presence] in Specimen by NAA with probe detection \| \|  \|  \| 97098-8 SARS-CoV-2 (COVID-19) Nsp2 gene [Presence] in Upper respiratory specimen by NAA with probe detection \| \|  \|  \| 96763-8 SARS-CoV-2 (COVID-19) E gene [Presence] in Respiratory specimen by NAA with probe detection \| \|  \|  \| 94316-7 SARS-CoV-2 (COVID-19) N gene [Presence] in Specimen by NAA with probe detection \| \|  \|  \| 94314-2 SARS-CoV-2 (COVID-19) RdRp gene [Presence] in Specimen by NAA with probe detection \| \|  \|  \| 95411-5 SARS-CoV-2 (COVID-19) neutralizing antibody [Presence] in Serum by pVNT \| \|  \|  \| 94762-2 SARS-CoV-2 (COVID-19) Ab [Presence] in Serum or Plasma by Immunoassay \| \|  \|  \| 95209-3 SARS-CoV+SARS-CoV-2 (COVID-19) Ag [Presence] in Respiratory specimen by Rapid immunoassay \| \|  \|  \| 100383-9 Monkeypox virus DNA [Presence] in Specimen by NAA with probe detection \| \|  \|  \| 80825-3 Zika virus envelope E gene [Presence] in Serum by NAA with probe detection \| \|  \|  \| 79190-5 Zika virus RNA [Presence] in Specimen by NAA with probe detection \| \|  \|  \| 87622-7 Zika virus IgM Ab [Presence] in Specimen by Immunoassay \| \| Value set \| 260373001 Detected (qualifier value) \| C25481 Detected \| LA11882-0 Detected \| \| 260415000 Not detected (qualifier value) \| C191300 Not Detected \| LA11883-8 Not detected \| \| 442754001 Inconclusive evaluation finding (finding) \| C48658 Indeterminate \|  \| \| 419984006 Inconclusive (qualifier value) \| C38758 Positive Finding \|  \| \| 10828004 Positive (qualifier value) \| C38757 Negative Finding \|  \| \| 897034005 Severe acute respiratory syndrome coronavirus 2 antibody test positive (finding) \| C48658 Indeterminate \|  \| \| 260385009 Negative (qualifier value) \| C191302 Weakly Positive \|  \| \| 442754001 Inconclusive evaluation finding (finding) \|  \|  \| \| 419984006 Inconclusive (qualifier value) \|  \|  \| \| 260408008 Weakly positive (qualifier value) \|  \|  \| |

| Table S5: List of concepts submitted for semantic standard code creation to NCIt and LOINC. |
| --- |
| \| **Suggested New Term** \| **SDO of submission** \| \| --- \| --- \| \| Sequencing date \| NCIt \| \| ZIKA virus serology sample collection date \| NCIt \| \| ZIKA virus PCR sample collection date \| NCIt \| \| Pangolin database version \| NCIt \| \| GISAID Accession ID \| NCIt \| \| SARS-CoV-2 PCR Date \| NCIt \| \| Anti-IFN antibody test date \| NCIt \| \| SARS-CoV-2 serology test date \| NCIt \| \| Serum SARS-CoV-2 neutralization test date \| NCIt \| \| SARS Coronavirus 2 P.2 \| NCIt \| \| SARS Coronavirus 2 A.23.1 \| NCIt \| \| SARS Coronavirus 2 A.27 \| NCIt \| \| SARS Coronavirus 2 B.1.1.318 \| NCIt \| \| MPXV Clade I \| NCIt \| \| MPXV Clade IIa \| NCIt \| \| MPXV Clade IIb \| NCIt \| \| MPXV Clade II \| NCIt \| \| Zika virus African lineage \| NCIt \| \| Zika virus Asian lineage \| NCIt \| \| SARS-CoV-2 RdRp gene \| NCIt \| \| SARS-CoV-2 N gene \| NCIt \| \| SARS-CoV-2 E gene \| NCIt \| \| SARS-CoV-2 S gene \| NCIt \| \| SARS-CoV-2 M gene \| NCIt \| \| SARS-CoV-2 ORF1ab gene \| NCIt \| \| Zika NS5 gene \| NCIt \| \| Zika E gene \| NCIt \| \| Zika M gene \| NCIt \| \| Sequencing date \| LOINC \| \| Zika virus lineage [Identifier] in Isolate \| LOINC \| \| Monkeypox virus neutralizing antibody [Titer] in Specimen by Neutralization test \| LOINC \| \| Monkeypox virus IgG Ab [Titer] in Serum by Immunofluorescence \| LOINC \| \| Monkeypox virus IgM Ab [Titer] in Serum by Immunofluorescence \| LOINC \| |
