# Supplementary figures and images for: Creation of Standardized Common Data Elements for Diagnostic Tests in Infectious Disease Studies: Semantic and Syntactic Mapping
Source: J Med Internet Res. 2024 Jun 10;26:e50049. doi: 10.2196/50049 (PMC11196918; doi:10.2196/50049)

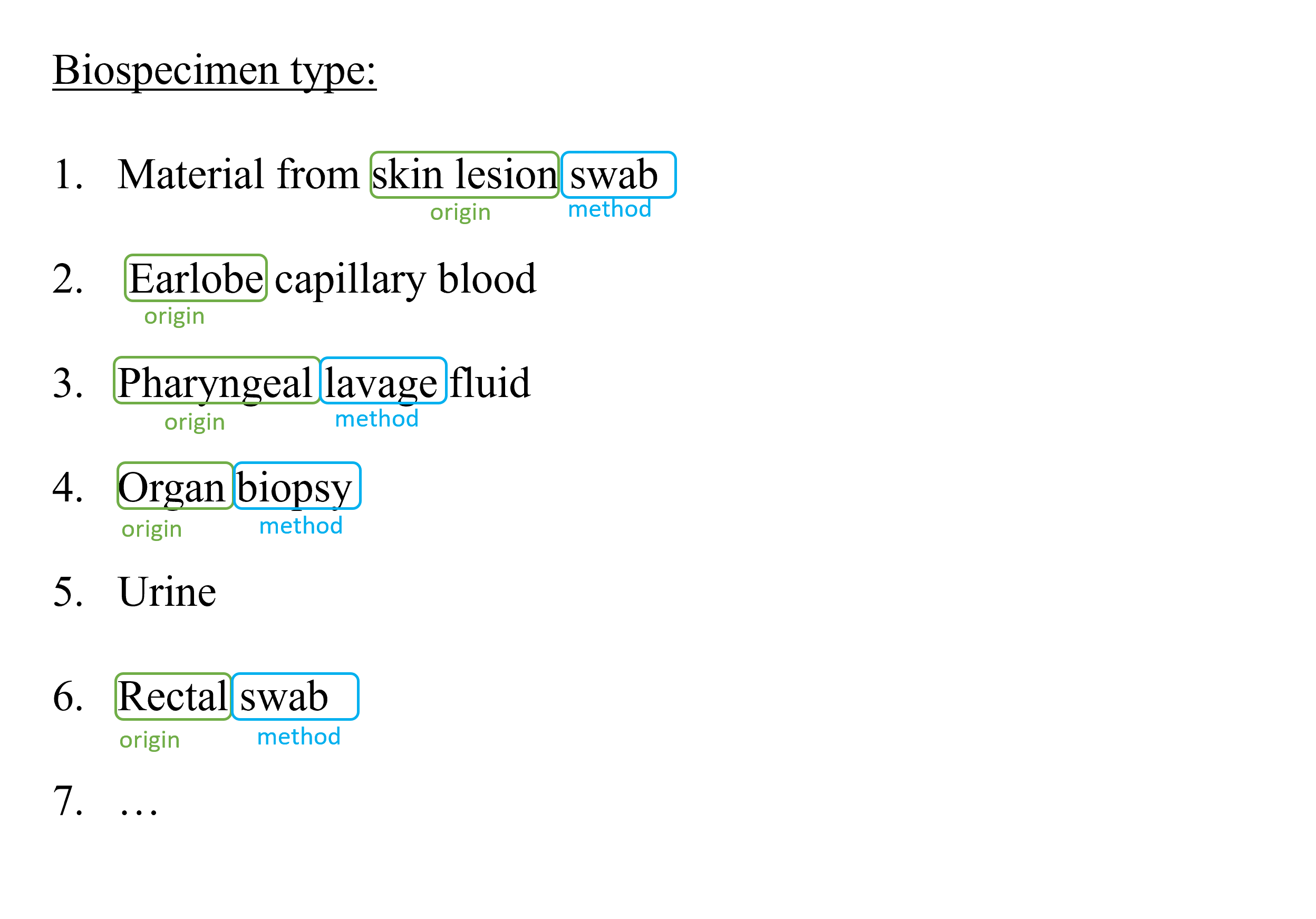

Supplement: Multimedia Appendix 3 [file jmir_v26i1e50049_app3.png]

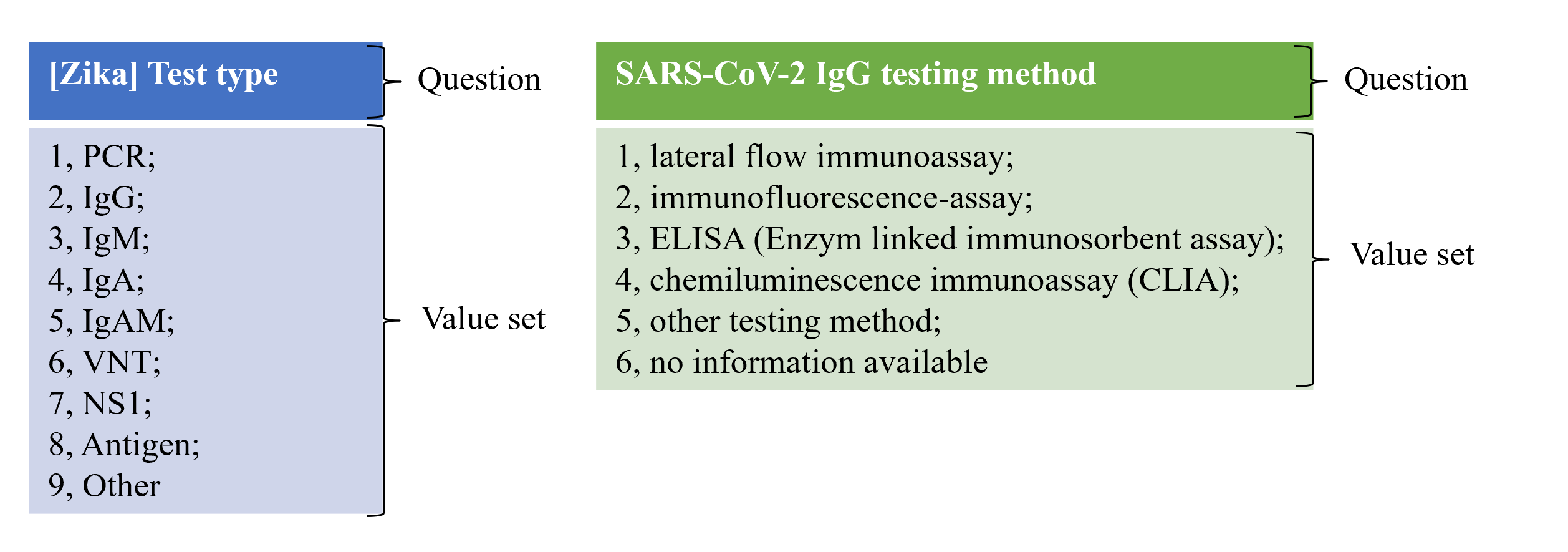

Supplement: Multimedia Appendix 4 [file jmir_v26i1e50049_app4.png]
